# Supplementary material for: Appressoria Formation in Phytopathogenic Fungi Suppressed by Antimicrobial Peptides and Hybrid Peptides from Black Soldier Flies
Source: Genes (Basel). 2023 May 17;14(5):1096. doi: 10.3390/genes14051096 (PMC10217845; doi:10.3390/genes14051096)
Supplement: Supplementary file 1 [file genes-14-01096-s001.zip › genes-2298069-supplementary.pdf]

Figure S1 Locations of the 34 genes encoding antimicrobial peptides in chromosome

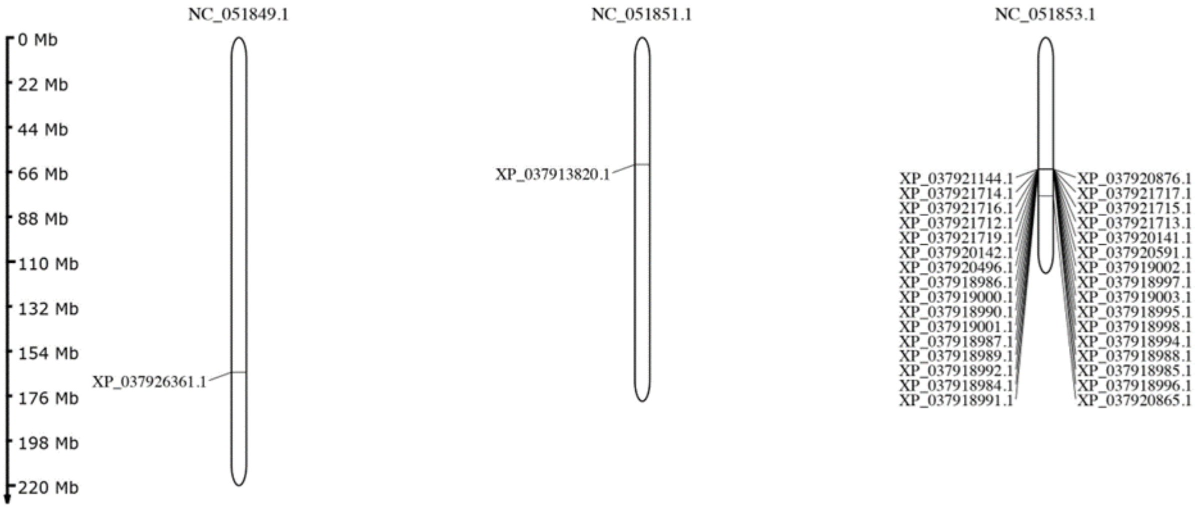

Figure S2 Mass spectrogram results of seven synthesized CAD peptides

生工® Sangon Biotech

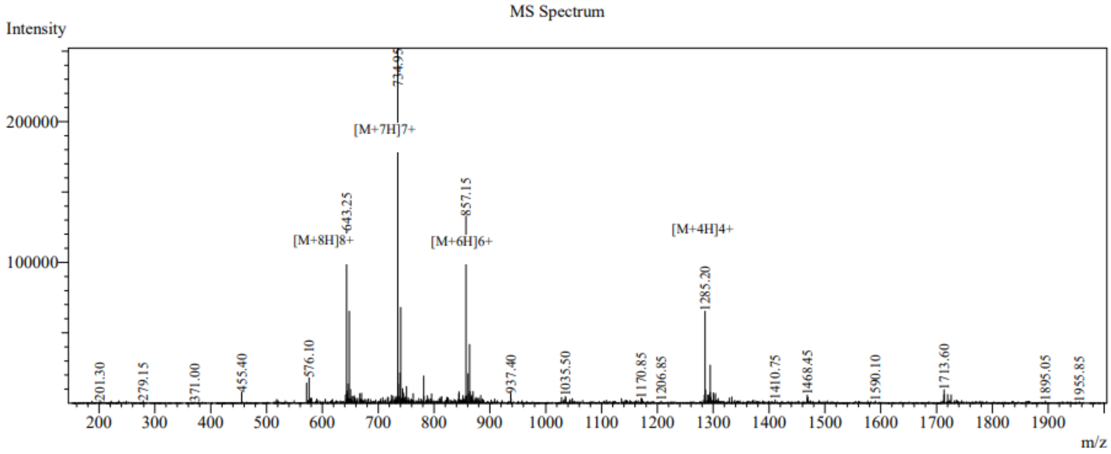

Sample Information
Dissolution method :0.1mg sample dissolved to 0.5mL by 50%ACN and 50%H2O
Date Acquired :2023/02/03 15:38:24
Injection Volume :1ul

Interface :ESI
Nebulizing Gas Flow :1.50L/min
CDL Temp :250°C
CDL Volt :0v
Block Temp :200

Prerod Bias :+4.5kv
Detector :-0.2kv
T.Flow :0.2ml/min
B.conc :50%H2O/50%MeOH

Name :CAD-1
Sequence :SLWKKLFKPVERAGQRIRDATIKGIAIAQQGANVLATVRGGPAIPPGQG
Modification :N/A
Lot No. :CAD-1-20230203
Theoretical :5137.007
bserved :5137.65

生工生物工程（上海）股份有限公司

地址：上海市松江区香闵路698号
电话/Tel: 400-821-0268
邮箱/Email: Sales@sangon.com

Add: 698 Xiang Min Road Songjiang Shanghai China
传真/Fax: 86-21-37772170
网址/Web: wwww.sangon.com

生工® Sangon Biotech

MS Spectrum

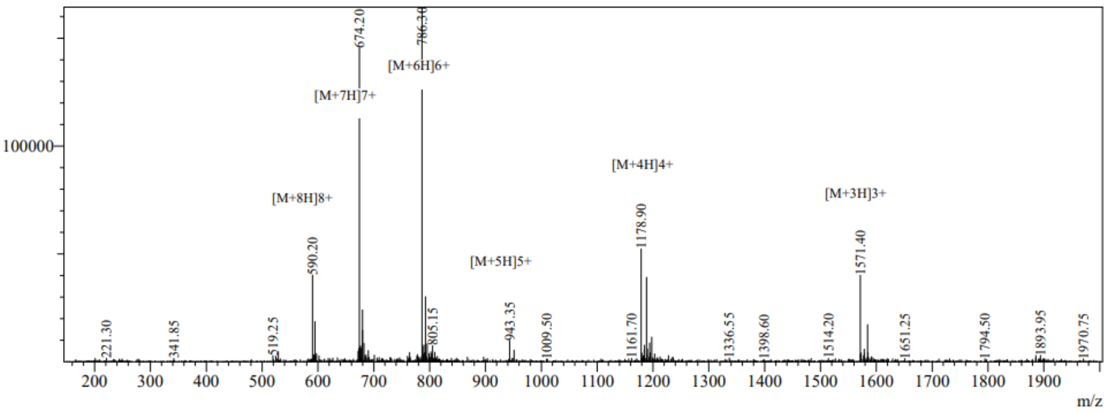

Sample Information
Dissolution method :0.1mg sample dissolved to 0.5mL by 50%ACN and 50%H2O
Date Acquired :2023/02/03 15:41:17
Injection Volume :1ul

Interface :ESI
Nebulizing Gas Flow :1.50L/min
CDL Temp :250℃
CDL Volt :0v
Block Temp :200

Prerod Bias :+4.5kv
Detector :-0.2kv
T.Flow :0.2ml/min
B.conc :50%H2O/50%MeOH

Name :CAD-2
Sequence :SLWKKIFKPVEKLGQRVRDAGIQGIAIAQQGANVLATIRGGPPQ
Modification :N/A
Lot No. :CAD-2-20230203
heoretical :4711.518
bserved :4711.80

生工生物工程（上海）股份有限公司

地址：上海市松江区香闵路698号
电话/Tel: 400-821-0268
邮箱/Email: Sales@sangon.com

Add: 698 Xiang Min Road Songjiang Shanghai China
传真/Fax: 86-21-37772170
网址/Web: wwww.sangon.com

生工® Sangon Biotech

MS Spectrum

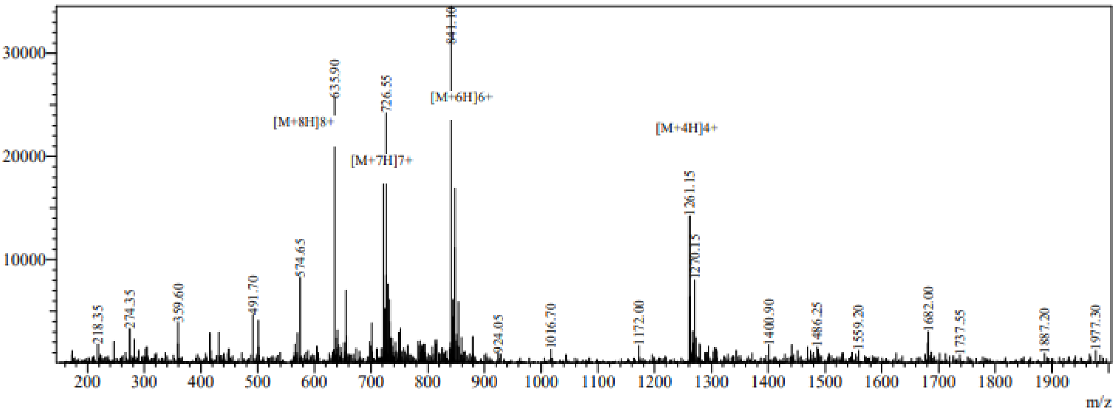

Sample Information
Dissolution method :0.1mg sample dissolved to 0.5mL by 50%ACN and 50%H2O
Date Acquired :2023/02/03 15:45:56
Injection Volume :1ul

Interface :ESI
Nebulizing Gas Flow :1.50L/min
CDL Temp :250℃
CDL Volt :0v
Block Temp :200

Prerod Bias :+4.5kv
Detector :-0.2kv
T.Flow :0.2ml/min
B.conc :50%H2O/50%MeOH

Name :CAD-3
Sequence :GWWKRVFKPVERLGQRVRDAGIQGLQIAQQGANVLATVRGGPLQQG
Modification :N/A
Lot No. :CAD-3-20230203
Theoretical :5040.805
bserved :5040.60

生工生物工程（上海）股份有限公司

地址：上海市松江区香闵路698号
电话/Tel: 400-821-0268
邮箱/Email: Sales@sangon.com

Add: 698 Xiang Min Road Songjiang Shanghai China
传真/Fax: 86-21-37772170
网址/Web: wwww.sangon.com

生工® Sangon Biotech

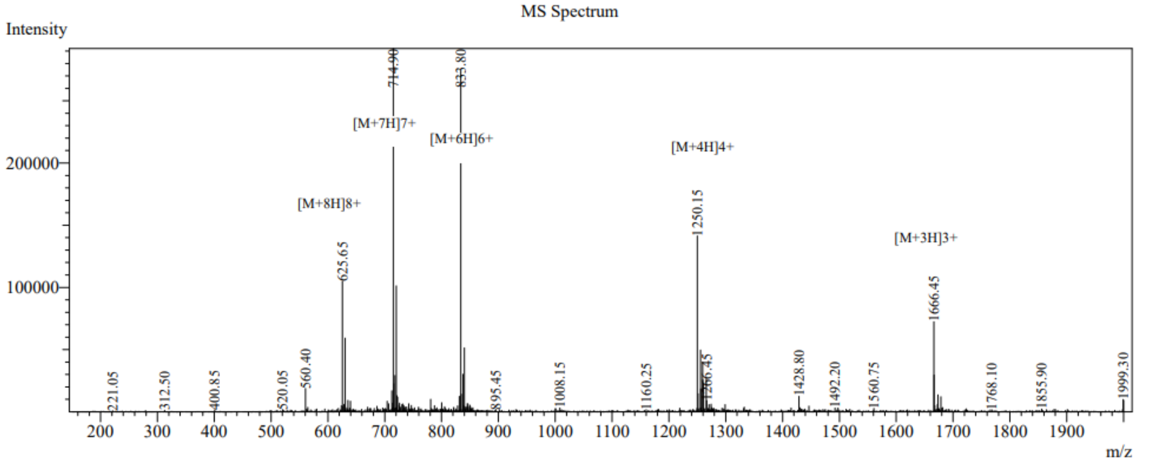

Sample Information
Dissolution method :0.1mg sample dissolved to 0.5mL by 50%ACN and 50%H2O
Date Acquired :2023/02/03 15:55:36
Injection Volume :1ul

Interface :ESI
Nebulizing Gas Flow :1.50L/min
CDL Temp :250°C
CDL Volt :0v
Block Temp :200

Prerod Bias :+4.5kv
Detector :-0.2kv
T.Flow :0.2ml/min
B.conc :50%H2O/50%MeOH

Name :CAD-7
Sequence :GWWKRVFKPVEKLGQRVRDAGIQGLQIAQQGANVLATVRGGPPQQG
Modification :N/A
Lot No. :CAD-7-20230203
Theoretical :4996.744
bserved :4997.20

生工生物工程（上海）股份有限公司

地址：上海市松江区香闵路698号
电话/Tel: 400-821-0268
邮箱/Email: Sales@sangon.com

Add: 698 Xiang Min Road Songjiang Shanghai China
传真/Fax: 86-21-37772170
网址/Web: wwww.sangon.com

生工® Sangon Biotech

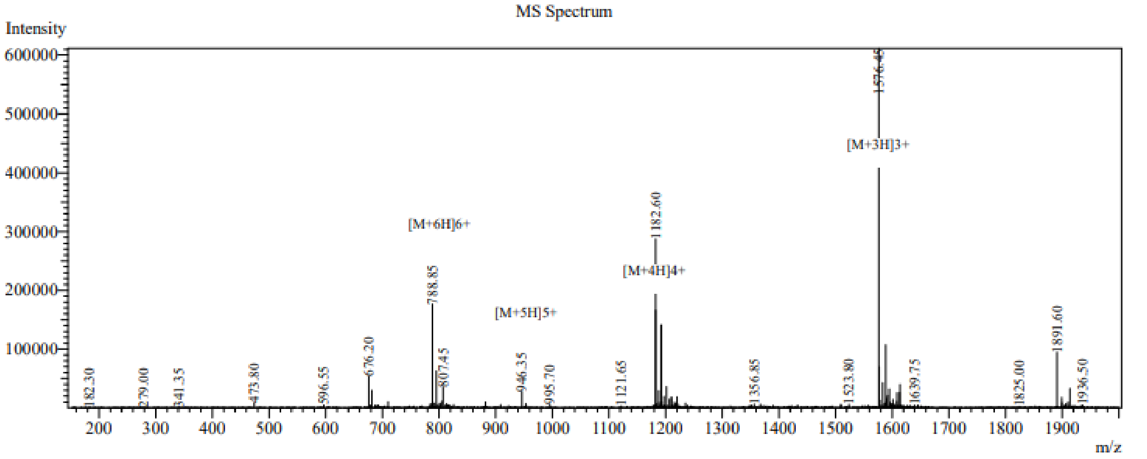

Sample Information
Dissolution method :0.1mg sample dissolved to 0.5mL by 50%ACN and 50%H2O
Date Acquired :2023/02/03 15:50:16
Injection Volume :1ul

Interface :ESI
Nebulizing Gas Flow :1.50L/min
CDL Temp :250°C
CDL Volt :0v
Block Temp :200

Prerod Bias :+4.5kv
Detector :-0.2kv
T.Flow :0.2ml/min
B.conc :50%H2O/50%MeOH

Name :CAD-5
Sequence :GWWKKVFKPVEKLGQRVRDAGIQGIAIAQQGANVLATVRGGPPQ
Modification :N/A
Lot No. :CAD-5-20230203
heoretical :4726.491
bserved :4726.35

生工生物工程（上海）股份有限公司

地址：上海市松江区香闵路698号
电话/Tel: 400-821-0268
邮箱/Email: Sales@sangon.com

Add: 698 Xiang Min Road Songjiang Shanghai China
传真/Fax: 86-21-37772170
网址/Web: wwww.sangon.com

生工® Sangon Biotech

MS Spectrum

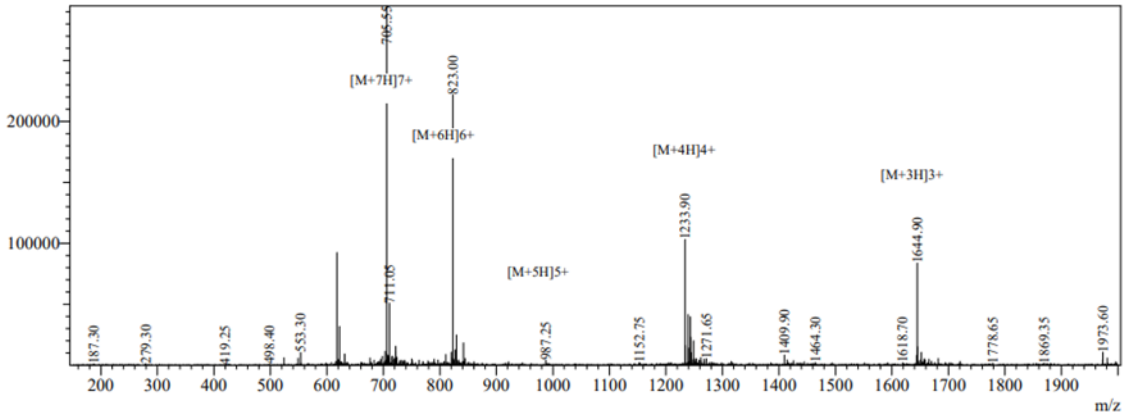

Sample Information
Dissolution method :0.1mg sample dissolved to 0.5mL by 50%ACN and 50%H2O
Date Acquired :2023/02/03 15:53:55
Injection Volume :1ul

Interface :ESI
Nebulizing Gas Flow :1.50L/min
CDL Temp :250℃
CDL Volt :0v
Block Temp :200

Prerod Bias :+4.5kv
Detector :-0.2kv
T.Flow :0.2ml/min
B.conc :50%H2O/50%MeOH

Name :CAD-6
Sequence :GWWKRVFKPVEKFGQRVRDAGVQGIAIAQQGANVLATARGGPPQQG
Modification :N/A
Lot No. :CAD-6-20230203
Theoretical :4931.628
bserved :4931.70

生工生物工程（上海）股份有限公司

地址：上海市松江区香闵路698号
电话/Tel: 400-821-0268
邮箱/Email: Sales@sangon.com

Add: 698 Xiang Min Road Songjiang Shanghai China
传真/Fax: 86-21-37772170
网址/Web: wwww.sangon.com

生工® Sangon Biotech

MS Spectrum

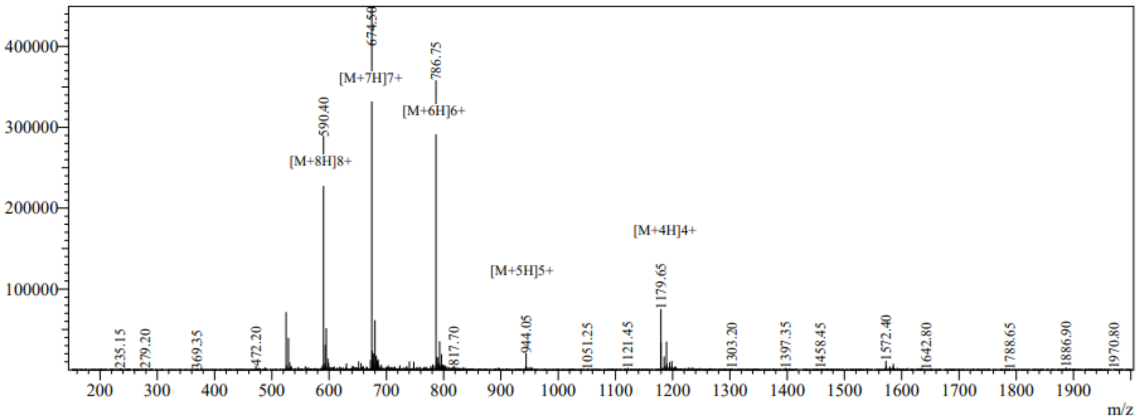

Sample Information
Dissolution method :0.1mg sample dissolved to 0.5mL by 50%ACN and 50%H2O
Date Acquired :2023/02/03 15:48:13
Injection Volume :1ul

Interface :ESI
Nebulizing Gas Flow :1.50L/min
CDL Temp :250℃
CDL Volt :0v
Block Temp :200

Prerod Bias :+4.5kv
Detector :-0.2kv
T.Flow :0.2ml/min
B.conc :50%H2O/50%MeOH

Name :CAD-4
Sequence :SWFKKVFKPVEKVGQRVRDAGIQGLTIAQQGANVLATARGGPPH
Modification :N/A
Lot No. :CAD-4-20230203
heoretical :4714.436
bserved :4714.50

生工生物工程（上海）股份有限公司

地址：上海市松江区香闵路698号
电话/Tel: 400-821-0268
邮箱/Email: Sales@sangon.com

Add: 698 Xiang Min Road Songjiang Shanghai China
传真/Fax: 86-21-37772170
网址/Web: www.sangon.com

Table S1 The Rename of the BSF AMPs.

GenBank	Sequence	Rename
XP 037921713.1	SLWKKLFKPVERAGQRIRDATIKGIAIAQQGANVLATVRGGPAIPPGQG	CAD1
XP 037921717.1	SLWKKIFKPVEKLGQRVRDAGIQGIAIAQQGANVLATIRGGPPQ	CAD2
XP 037918997.1	GWWKRVFKPVERLGQRVRDAGIQGLQIAQQGANVLATVRGGPLQQG	CAD3
XP 037920142.1	SWFKKVFKPVEKVGQRVRDAGIQGLTIAQQGANVLATARGGPPH	CAD4
XP 037920591.1	GWWKKVFKPVEKLGQRVRDAGIQGIAIAQQGANVLATVRGGPPQ	CAD5
XP 037918984.1	GWWKRVFKPVEKFGQRVRDAGVQGIAIAQQGANVLATARGGPPQQG	CAD6
XP 037918991.1	GWWKRVFKPVEKLGQRVRDAGIQGLQIAQQGANVLATVRGGPPQQG	CAD7

Table S2 Basic characterization data of seven mature CAD peptides

Name	Number of amino acids	Molecular weight	Theoretical pI	Hydrophilicity
CAD1	49	5.137 KDa	11.58	hydrophobic
CAD2	44	4.711 KDa	11.07	hydrophobic
CAD3	46	5.04 KDa	11.83	hydrophobic
CAD4	44	4.714 KDa	11.07	hydrophobic
CAD5	44	4.726 KDa	11.07	hydrophobic
CAD6	46	4.931 KDa	11.57	hydrophobic
CAD7	46	4.996 KDa	11.57	hydrophobic

Table S3 Oligonucleotide sequences used in this study

**Primer Name**	**Primer Sequence (5′→3′)**	**Primer length (bp)**
Actin-F	CAACGAGCGATTCAGGTGTCC	21
Actin-R	GAGTGCGGTGATTTCCTTCTGC	22
CAD1-F	ATGAATTTCTCAAAGCTTCTCATCG	25
CAD1-R	TTATCCTTGTCCGGGGGGAATTGCT	25
CAD5-F	ATGAATTTCACTAAACTTTTTGTTG	25
CAD5-R	TTATTGTGGTGGTCCACCTCGAACC	25
CAD7-F	ATGAACTTCACAAAGCTTTTCGTCAT	26
CAD7-R	TTATCCTTGTTGGGGTGGTCCACCT	25
Actin-qRT-F	CAACGAGCGATTCAGGTGT	19
Actin-qRT-R	GAGTGCGGTGATTTCCTTCT	20
CAD1-qRT-F	ATGAATTTCTCAAAGCTTC	19
CAD1-qRT-R	TTATCCTTGTCCGGGGGGAA	20
CAD2-qRT-F	ATGAACTTCACAAAGCTTT	19
CAD2-qRT-R	TTATTGTGGTGGGCCTCCTC	20
CAD5-qRT-F	ATGAATTTCACTAAACTTT	19
CAD5-qRT-R	TTATTGTGGTGGTCCACCTC	20
CAD3-qRT-F	ATGAATTCCGCAAAGCTTT	20
CAD3-qRT-R	TTATCCTTGTTGGAGTGGTC	20
CAD7-qRT-F	ATGAACTTCACAAAGCTTT	19
CAD7-qRT-R	TTATCCTTGTTGGGGTGGTC	20

Table S4 Amino acid sequence of CAD-Con.

GWWKKVFKPVEKLGQRVRDAGIQGIAIAQQGANVLATVRGGPPQGWWKRVFKPVEKLGQRVRDAGIQGLQIAQQGANVLATVRGGPPQQGSLWKKLFKPVERAGQRIRDATIKGIAIAQQGANVLATVRGGPAIPPGQG
